# Metabolic Encephalopathy and Lipid Storage Myopathy Associated with a Presumptive Mitochondrial Fatty Acid Oxidation Defect in a Dog

**DOI:** 10.3389/fvets.2015.00064

**Published:** 2015-11-26

**Authors:** Vanessa R. Biegen, John P. McCue, Taryn A. Donovan, G. Diane Shelton

**Affiliations:** ^1^The Animal Medical Center, New York, NY, USA; ^2^The Department of Pathology, School of Medicine, University of California San Diego, La Jolla, CA, USA

**Keywords:** metabolic encephalopathy, lipid storage myopathy, inborn error of metabolism, fatty acid oxidation, magnetic resonance imaging

## Abstract

A 1-year-old spayed female Shih Tzu presented for episodic abnormalities of posture and mentation. Neurological examination was consistent with a bilaterally symmetric multifocal encephalopathy. The dog had a waxing-and-waning hyperlactemia and hypoglycemia. Magnetic resonance imaging revealed bilaterally symmetric cavitated lesions of the caudate nuclei with less severe abnormalities in the cerebellar nuclei. Empirical therapy was unsuccessful, and the patient was euthanized. Post-mortem histopathology revealed bilaterally symmetric necrotic lesions of the caudate and cerebellar nuclei and multi-organ lipid accumulation, including a lipid storage myopathy. Malonic aciduria and ketonuria were found on urinary organic acid screen. Plasma acylcarnitine analysis suggested a fatty acid oxidation defect. Fatty acid oxidation disorders are inborn errors of metabolism documented in humans, but poorly described in dogs. Although neurological signs have been described in humans with this group of diseases, descriptions of advanced imaging, and histopathology are severely lacking. This report suggests that abnormalities of fatty acid metabolism may cause severe, bilateral gray matter necrosis, and lipid accumulation in multiple organs including the skeletal muscles, liver, and kidneys. Veterinarians should be aware that fatty acid oxidation disorders, although potentially fatal, may be treatable. A timely definitive diagnosis is essential in guiding therapy.

## Case Presentation

A 1-year-old spayed female Shih Tzu was evaluated at The Animal Medical Center for several episodes of dull mentation, disorientation, and difficulty walking. At 4 months of age, the dog presented to another hospital for acute hypertonic non-ambulatory tetraparesis, which improved quickly to a non-ambulatory paraparesis, and diffuse generalized tremors. Neurological abnormalities included proprioceptive deficits in all limbs and a positional vertical nystagmus. Complete blood count, serum chemistry panel, pre- and postprandial bile acids, abdominal ultrasound, thoracic and abdominal radiographs, and a panel screening for tick-borne diseases utilizing polymerase chain reaction^1^ failed to identify any significant abnormalities. The dog was treated supportively with intravenous fluids and released the following day when clinical signs resolved. The dog had three additional episodes over the following 8 months, all with spontaneous improvement within hours. All episodes were associated with perceived stressors, such as holidays and the presence of visitors in the house. The dog was reportedly normal between episodes. Two days prior to presentation, the dog was found in dorsal recumbency with pelvic limb extensor rigidity and an abnormal mentation. Resolution occurred within hours. The following 2 days, the dog had multiple episodes of heavy panting and abnormal mentation lasting several hours, followed by an acute onset of non-ambulatory tetraparesis and disorientation just prior to presentation.

On presentation, the dog had a rectal temperature of 103.1°F and a systolic blood pressure of 220 mmHg, both of which returned to normal within hours following initial stabilization and therapy with hypertonic saline and replacement crystalloid intravenous fluids. Physical examination was otherwise normal. Abnormalities on neurological examination included a dull mentation, fine intermittent head tremors, non-ambulatory tetraparesis with absent postural reactions in all limbs, absent menace responses bilaterally, and a bilateral positional ventrolateral strabismus. Muscle tone and spinal reflexes were normal. These findings were consistent with a bilaterally symmetric multifocal encephalopathy with differential diagnoses including congenital (e.g., hydrocephalus), toxic (e.g., lead), metabolic (e.g., hepatic encephalopathy, inborn error of metabolism), neurodegenerative (e.g., idiopathic superficial neocortical degeneration and atrophy of young dogs), nutritional (e.g., thiamine deficiency), and viral etiologies. Metabolic encephalitides were prioritized due to the episodic nature of the signs. A venous blood gas and electrolyte panel revealed a mild hyperlactemia (2.63 mmol/L; reference interval, 0.5–2.5 mmol/L) and mild hypocapnia (32.9 mmHg; reference interval 35–45 mmHg); pH was 7.4 (reference interval, 7.35–7.45) and bicarbonate was 19.9 mmol/L (reference interval, 18–24 mmol/L). The dog was treated with a bolus of 3% hypertonic saline (5.3 mL/kg IV), maropitant citrate (1 mg/kg IV q 24 h), pantoprazole (1 mg/kg IV q 24 h), and a replacement crystalloid^2^(4 mL/kg/h) supplemented with potassium chloride (20 meq/L). After an overnight fast, the patient had a worsened mentation and a serum lactate of 13.2 mmol/L. The dog became hypertensive with a blood pressure of 186/112 mmHg and developed a sinus tachycardia of 175 bpm. Boluses of the replacement crystalloid (see text footnote 2) (22 mL/kg IV) and 3% hypertonic saline (5.3 mL/kg IV) were administered. Lactate was noted to improve 5.77 mmol/L following these interventions, and the dog’s mentation transiently improved.

**Figure 1:**
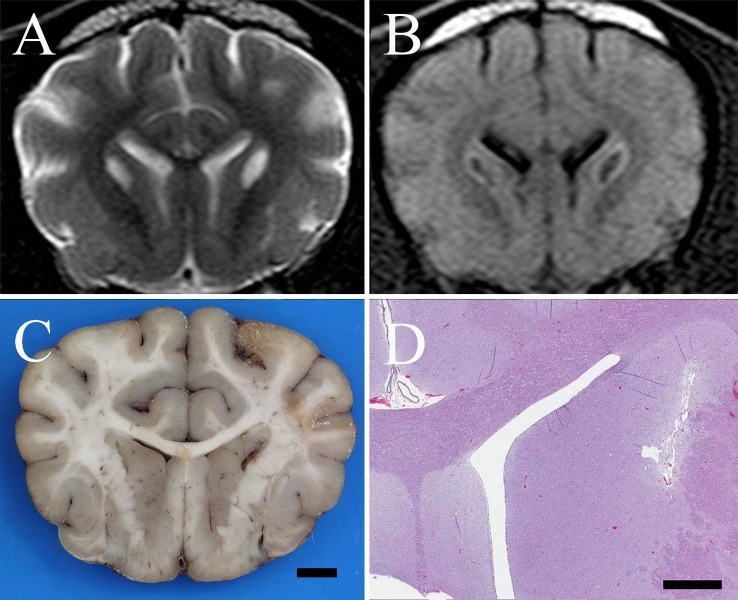
**(A)** Transverse T2-weighted turbo spin echo MR image of a 1-year-old spayed female Shih Tzu presenting for episodic multifocal encephalopathy. There are bilaterally symmetric, teardrop-shaped hyperintensities of the caudate nuclei suggestive of a metabolic encephalopathy. **(B)** Transverse FLAIR MR image at the same level as in **(A)** reveals the caudate nuclear lesions are hypointense with hyperintense rims. This suggests a low-protein cavitated lesion within the caudate nuclei surrounded by either edema, proteinaceous fluid, inflammation, or, less likely, neoplastic tissue. **(C)** Formalin fixed brain, dog. Photograph of a transverse section of the cerebrum at the level of the caudate nuclei. There are bilateral, linear cavitated foci of necrosis within the caudate nuclei adjacent to the internal capsule; bar = 5 mm. **(D)** Brain, dog. Photomicrograph at the same level as **(C)**. Bilateral, linear, cavitated foci of necrosis are present adjacent to the internal capsule. H&E stain; Subgross Image, bar = 1.5 mm.

**Figure 2:**
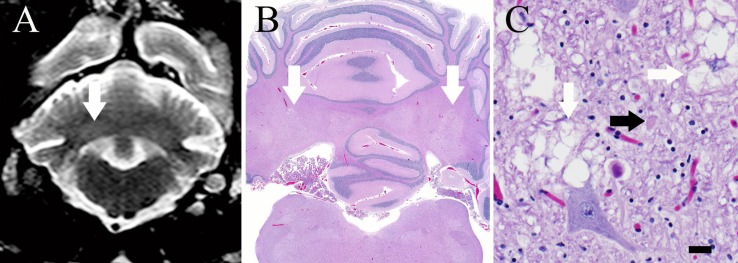
**(A)** Transverse T2-weighed turbo spin echo MR image at the level of the cerebellar nuclei. There are subtle, bilaterally symmetric hyperintensities of the cerebellar nuclei (white arrow) that correspond to areas of vacuolation on histopathology. **(B)** Brain, dog. Subgross photomicrograph of the cerebellum and medulla. Multifocal regions of pallor are present at the level of the lateral, interposital, and fastigial nuclei (white arrows), corresponding with the MRI. H&E stain. **(C)** Brain, dog. High magnification photomicrograph of the cerebellum within a pale region denoted by the arrows in **(B)**. Note the myelin vacuolation (white arrows) and axonal swelling (spheroid, black arrow). There are increased numbers of glial cells in this region, including microglia and astroglia, which are multifocally undergoing cytoplasmic swelling. H&E stain; 400 × magnification; bar = 20 *μ*m.

A resting ammonia was normal (5 *μ*mol/L; reference interval, 2–75 *μ*mol/L). Complete blood count and serum chemistry profile were recommended but declined by the owner. Cervical and brain MRI performed with a 1.5-T unit^3^ revealed non-contrast-enhancing bilaterally symmetric, teardrop-shaped T2-hyperintensities of the caudate nuclei that were hypointense with hyperintense periphery on FLAIR and hypointense to gray matter on T1-weighted images (Figures 1A,B). Non-contrast enhancing, bilaterally symmetric, subtle T2-hyperintensities of the cerebellar nuclei that remained hyperintense on FLAIR and isointense to gray matter on T1-weighted images were also identified (Figure 2A). Cerebrospinal fluid collected from the atlanto-occipital subarachnoid space had a normal nucleated cell count (1/*μ*L; reference interval, <4/*μ*L) and protein concentration (11 mg/dL; reference interval, <35 mg/dL); cytological examination disclosed primarily mature lymphocytes with occasional large monocytoid mononuclear cells. CSF lactate was also normal (1.2 mmol/L; reference interval, 0.416–1.850 mM/L) and was similar to that of a control dog measured the same day (1).

Based on the episodic, multifocal, and symmetrical clinical signs and the bilaterally symmetric gray matter lesions on MRI, etiological categories including toxic, metabolic, degenerative, and nutritional abnormalities were considered. The dog had no history of toxin exposure and was on a balanced commercial canine diet. A quantitative urine organic acid screen was submitted to Biochemical Genetics Laboratory at the University of California San Diego during a period of normoglycemia, the results of which were not available for several weeks. Intravenous replacement crystalloids (see text footnote 2) were continued with the addition of B-complex vitamins (2 mL/L of replacement crystalloid). Thiamine therapy was also instituted (11 mg/kg SQ q 12 h).

Over the following 3 days, there was no improvement in neurological status. The dog became intermittently hypoglycemic (as low as 50 mg/dL; reference interval, 62–114 mg/dL), which was responsive to feedings. Blood lactate varied between 1.05 and 4.62 mmol/L, often accompanied by panting and resultant hypocapnia, and when >2 mmol/L, was treated with fluid boluses in addition to maintenance fluids. On day 3, methylprednisolone sodium succinate (30 mg/kg IV) was administered and oral prednisone (0.54 mg/kg PO q 12 h) was initiated due to concerns regarding possible inflammation associated with the areas represented by hyperintense rings on FLAIR sequences. Levetiracetam (27 mg/kg PO q 8 h) was also initiated for its reported neuroprotective qualities (2). The patient was humanely euthanized on day 4 due to lack of improvement. Consent was obtained for post-mortem examination.

On post-mortem examination, gross abnormalities included bilateral cavitated foci within the caudate nuclei (Figure 1C), pallor of several focal skeletal muscle bundles, dorsal and pelvic limb muscle wasting, multifocal to coalescing hepatic parenchymal pallor, and congestion of multiple organs. Histopathological examination of the brain identified severe, bilateral focal necrosis of the caudate nuclei with cavitation, Gitter cell infiltration, rare axonal swelling (spheroids), regional rarefaction and gliosis (Figure 1D). In the cerebellum, neuropil vacuolation was present at the level of the lateral, interposital, and fastigial nuclei, accompanied by gliosis, glial cytoplasmic swelling, and axonal spheroid formation (Figures 2B,C). There was also regional granular cell layer depletion within the cerebellar cortex. Within the brainstem, mild vacuolation was found bilaterally in the region of the vestibular and cuneate nuclei. In all segments of the spinal cord, there was mild, multifocal myelin vacuolation with myelinophages and mild multifocal gray matter gliosis. Histologically examined skeletal muscles multifocally contained small, discrete, well demarcated, circular, and coalescing vacuoles that were morphologically consistent with lipid. In the kidneys, vacuoles within proximal and distal tubules were well demarcated, discrete, and circular, consistent with lipidosis. Diffusely throughout hepatic lobules, but more prominently in periportal regions, hepatocytes contained multiple, round, and discrete vacuoles, which did not displace the nucleus (microvesicular lipidosis).

**Figure 3:**
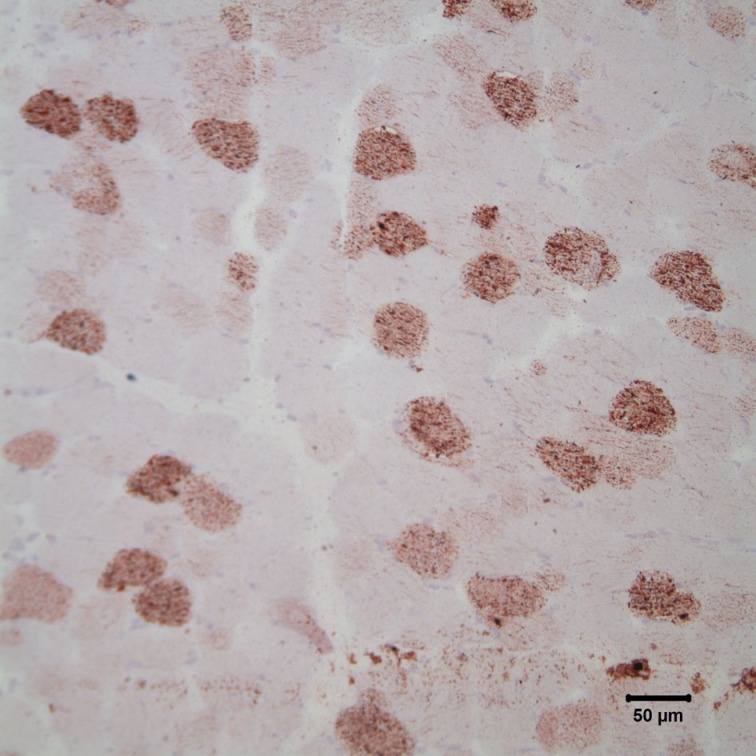
**Photomicrograph of cryosections of the triceps muscle stained with oil red O stain reveals the presence of numerous intramyofiber lipid droplets, consistent with a lipid storage myopathy**. Oil red O stain; bar = 50 *μ*m.

Fresh and fixed biopsies of the left adductor, triceps brachii, vastus lateralis, and obliquus capitis cranialis muscles were submitted under refrigeration to the Comparative Neuromuscular Laboratory at the University of California San Diego. The unfixed samples were evaluated in frozen sections and the fixed samples were evaluated in paraffin. A standard panel of histological and histochemical stains and reactions were employed including hematoxylin and eosin, modified Gomori trichome, periodic acid-Schiff, myofibrillar adenosine triphosphatase reactions at pH 9.8 and 4.3, esterase, reduced nicotinamide adenine dinucleotide-tetrazolium reductase, alkaline phosphatase, acid phosphatase, oil red O, and Staphylococcal protein A-horseradish peroxidase. The most striking abnormality was the presence of numerous vacuoles within most myofibers containing excessive and variably sized intramyofiber lipid droplets utilizing the oil red O stain for neutral triglycerides (Figure 3). Findings were consistent with a lipid storage myopathy associated with a fatty acid oxidation defect (FAOD) or a primary or secondary disorder of carnitine metabolism.

Quantitative urine organic acids screening was performed in the Biochemical Genetics Laboratory at the University of California San Diego using gas chromatography-mass spectrometry, as previously described (3). Testing revealed moderate urinary excretion of malonic acid (41 mmol/mol creatinine; reference interval, 0–1 mmol/mol creatinine), lactic acid (256 mmol/mol creatinine; reference interval, 0–200 mmol/mol creatinine), the ketones 3OH^−^ butyric acid (1109 mmol/mol creatinine; reference interval, 0–11 mmol/mol creatinine), acetoacetic acid (275 mmol/mol creatinine; reference interval, 0–1 mmol/mol creatinine), suberic acid (30 mmol/mol creatinine; reference interval, 0–4 mmol/mol creatinine), and hexanoylglycine (10 mmol/mol creatinine; reference interval, <2 mmol/mol creatinine). This pattern of abnormalities suggested a FAOD such as medium-chain acyl-CoA dehydrogenase deficiency (MCADD) (4, 5).

For further investigation, plasma acylcarnitine analysis was performed and revealed elevated C14:1 (0.26 *μ*M; reference interval, 0–0.2 *μ*M) with an elevated C14:C8:1 ratio (14.51; reference interval, <1.25), an elevated C16:1 (0.21 *μ*M; reference interval, 0–0.07 *μ*M) with an elevated C16/C8.1 ratio (16.61; reference interval <1.7) and elevated C18.1 (0.41 *μ*M; reference interval, 0–0.24 *μ*M) with an elevated C18.1/C8.1 ratio (23.17 reference interval >1.7) (6). These compounds are representative of long-chain fatty acids (LCFAs) 14–18 carbons in length. This pattern suggests a long-chain FAOD such as very long-chain acyl-CoA dehydrogenase deficiency (VLCADD) or carnitine palmitoyltransferase-2 (CPT2) deficiency. The C3DC (malonyl)-carnitine was not elevated, ruling out a primary malonic aciduria (5, 7).

## Background

Organic acidurias (OAs) and mitochondrial FAODs are inborn errors of metabolism that have been described rarely in the veterinary literature. OAs involve defects in the metabolic pathways of carbohydrates, proteins, or fats. For example, l-2 hydroxyglutaric aciduria, one of the most well described OAs in the veterinary literature, involves a defect in the enzyme that converts l-2 hydroxyglutarate into 2-oxoglutarate, a citric acid cycle intermediate and glutamate metabolite (8). In addition, there have been single reports of malonic aciduria in a family of Maltese dogs (9) and a combined malonic and methylmalonic aciduria in a Labrador retriever (10). Mitochondrial FAODs, more specifically, involve a deficiency of one or more enzymes involved in the transport and β-oxidation of fatty acids, decreasing the ability of cells to use fat for energy production and leading to the accumulation of substrates upstream from the defect. These include deficiencies in CPTs (involved in the uptake of fatty acids by the mitochondria) and in acyl-CoA dehydrogenases (involved in the β-oxidation of fatty acids) (11). Veterinary reports include that of a dog with refractory seizures secondary to MCADD (12) and a report of two horses with rhabdomyolysis secondary to multiple acyl-CoA dehydrogenase deficiency (MADD) (13).

## Discussion

**Figure 4:**
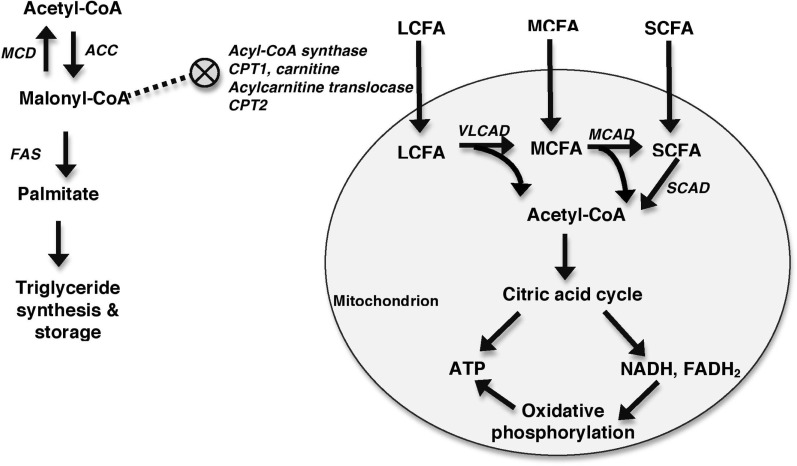
**Generalized schematic depicting mitochondrial FAO**. Fatty acids enter the mitochondria with MCFAs and SCFAs diffusing in and LCFAs requiring the assistance of a number of molecules including acyl-CoA synthase, CPT1, carnitine, acylcarnitine translocase, and CPT2 to enter the mitochondria. This process is inhibited by malonyl-CoA, which inhibits CPT1. Malonyl-CoA is primarily formed from acetyl-CoA in a reaction catalyzed by acetyl-CoA carboxylase (ACC). Malonyl-CoA can be metabolized back into acetyl-CoA by MCD. The roles of malonyl-CoA include the synthesis and elongation of fatty acids in reactions catalyzed by fatty acid synthase (FAS), and the inhibition of CPT1. Once within the mitochondria, fatty acids undergo β-oxidation to form acetyl-CoA. VLCAD oxidizes LCFAs, MCAD oxidizes MCFAs, and short-chain acyl-CoA dehydrogenase (SCAD) oxidizes SCFA. Acetyl-CoA is then shuttled into the citric acid cycle for production of ATP and reducing coenzymes, such as NADH and FADH_2_. These reducing coenzymes can then be utilized in oxidative phosphorylation to produce additional ATP.

Metabolic profiling in the patient described in this report was consistent with a FAOD. FAO, or more specifically β-oxidation, occurs in the mitochondria and involves the breakdown of fatty acids into acetyl-CoA. Acetyl-CoA then acts as the primary substrate for the citric acid cycle, which produces ATP and the reducing coenzymes (e.g., NADH and FADH_2_) used in the electron transport chain (i.e., oxidative phosphorylation) for further ATP production. Acetyl-CoA can be formed from the breakdown of fats, glucose (via glycolysis), ketones, and proteins. In order for β-oxidation to occur, fatty acids must first enter the mitochondria (Figure 4). The ability of fatty acids to cross the mitochondrial membranes depends on the number of carbons in the chain. Medium-chain fatty acids (MCFAs) and short-chain fatty acids (SCFAs) are able to diffuse into the mitochondria without the use of a carrier molecule. LCFAs require the use of the transport molecules CPT1, CPT2, and acylcarnitine translocase in a stepwise process utilizing carnitine. β-oxidation involves the stepwise removal of two carbons at a time from the fatty acids to form molecules of acetyl-CoA. Each of these reactions is catalyzed by enzymes specific to the length of the molecule. For example, very long-chain acyl-CoA dehydrogenase will shorten very long-chain fatty acids (VLCFAs) and LCFAs into MCFAs, releasing molecules of acetyl-CoA in the process. Medium-chain acyl-CoA dehydrogenase, in turn, will shorten MCFAs into SCFAs in a process that also releases acetyl-CoA. A defect at any level in this process may lead to an impaired ability to utilize energy from fats (11, 14, 15).

In addition to entering the citric acid cycle, acetyl-CoA also stimulates gluconeogenesis and is used in the formation of ketones and malonyl-CoA (14, 15). Malonyl-CoA plays two major roles in the fed state in order to promote fat storage over β-oxidation. In a series of reactions catalyzed by the fatty acid synthase complex, it is used to form LCFAs, and eventually triglycerides, in adipogenic tissue (e.g., liver and adipocytes) (16). In addition, it inhibits CPT1, preventing the transport of LCFAs across the mitochondrial membrane, which is the rate-limiting step in β-oxidation of LCFAs (17). During times of energy need, malonyl-CoA levels are decreased by malonyl-CoA decarboxylase (MCD), which metabolizes malonyl-CoA into acetyl-CoA and carbon dioxide, decreasing its inhibition of β-oxidation (16).

Fatty acid oxidation defects are poorly recognized and classified in the veterinary literature, and the specific enzymatic defect was not identified in this case. Enzymatic assays in cultured fibroblasts and gene sequencing would have been useful in definitive identification of the affected enzymes, and are widely utilized in people. While the elevated levels and proportions of long-chain acylcarnitines were most supportive of a VLCADD or CPT2 deficiency, the organic acid screen revealed a hexanoylglycinuria, which has been most frequently associated with MCADD and MADD (4, 5, 11). A moderate malonic aciduria was also identified on the urine organic acid screen, which, to the authors’ knowledge, has not been previously associated with a FAOD. Canine malonic aciduria has been described in a family of Maltese dogs with neurological signs (9), and in a Labrador retriever that also suffered from methylmalonic aciduria (10). As an acylcarnitine analysis was not performed in the report of Maltese dogs, it is unclear whether these dogs suffered from a primary malonic aciduria or some other inborn error of metabolism with a secondary malonic aciduria, as suspected in this patient. Primary malonic aciduria has been well characterized in the human literature and is most commonly associated with a deficiency of MCD, the enzyme that metabolizes malonyl-CoA. This diagnosis is confirmed by identifying elevated malonylcarnitine levels, reduced MCD levels in cultured fibroblasts, and genetic testing (5, 16, 17). This defect was considered unlikely in the patient of this report due to the normal malonylcarnitine levels. Clinical signs and laboratory abnormalities associated with malonic aciduria are very similar to those seen in this patient and overlap substantially with many of the FAODs (5, 9, 10, 16–18). This is likely due to the inhibitory effect that elevated levels of malonyl-CoA have on β-oxidation of fatty acids. Therapeutic strategies are also similar.

The phenotypes of OAs and FAODs are heterogenous and overlap substantially. Clinical signs and laboratory abnormalities are often episodic and may be induced by stressors such as fasting and infection (5, 11, 19, 20). The dog in this report had periods of hypoglycemia, hyperlactemia, and ketonuria, and clinical signs worsened with fasting, consistent with various OAs and FAODs described in the human literature. The hypoglycemia and hyperlactemia can be explained by an increased reliance upon glycolysis due to decreased availability of ATP from FAO, a lack of induction of gluconeogenesis by the products of β-oxidation, and on inhibition of enzymes by accumulated metabolites. While human FAODs are classically associated with a hypoketotic hypoglycemia, ketone levels may be variable in both pure FAODs and in FAODs that occur with other concurrent metabolic abnormalities (e.g., mitochondrial disorders) (5, 11, 15, 20). Ketogenesis requires initial β-oxidation, which was defective in this patient. The presence of ketones in this patient was likely due to the fact that the block in β-oxidation was incomplete and that dysfunction of β-oxidation can result in outflow of acetyl-CoA (the major substrate in ketogenesis) from other sources, such as ketogenic amino acids (14).

Reported clinical signs in the human and veterinary literature include encephalopathies, developmental delay, seizures, myopathic syndromes (including lipid storage myopathies and rhabdomyolysis), cardiomyopathies, hepatopathies, and sudden neonatal death (5, 11, 15, 19, 20). The major clinical signs of the dog in this report were initially episodic and were reflective of a bilaterally symmetric encephalopathy. The exact cause of intracranial changes in FAODs and OAs is unknown, but neonatal hypoglycemia, metabolic crises, lactic acidemia, disordered energy production, and direct toxic effects of accumulated metabolites have been proposed as potential mechanisms (20, 21). Brain MRI abnormalities described in the human literature are also heterogenous and dependent on the specific syndrome, the severity of signs, and the age of the affected individual. Abnormalities may include white matter changes, gray matter changes, atrophy, and/or evidence of neuronal migration defects (21, 22). Bilaterally symmetric basal ganglia lesions, as predominated in the dog in the case reported here, have been found in many of the inborn errors of metabolism affecting the brain, including FAODs, likely secondary to the highly metabolically active nature of these nuclei (21–23). A retrospective study examining MRI abnormalities after hypoglycemic crises in infants and children with inborn errors of metabolism, including FAODs, correlated basal ganglia lesions with hypoglycemia during the ages 6–22 months (23). Lesions of the caudate nuclei have been described in other metabolic disorders of dogs including l-2-hydroxyglutaric aciduria, GM2-gangliosidosis, and Alaskan Husky encephalopathy, as well as in a dog with recurrent hypoglycemia secondary to an insulinoma (8, 22–26).

On post-mortem examination, the dog described here had significant changes in a variety of organs. Lipid accumulation was identified in the liver, kidneys, and muscles. Lipid storage myopathy has been described in FAODs and disorders of carnitine metabolism and is likely secondary to an inability of the cells to properly metabolize fats (27). Urine organic acid analysis in a series of dogs with lipid storage myopathy supported an etiology of disorders of mitochondrial oxidative metabolism (28). In addition, the horses with MADD were found to have microvesicular lipidosis on muscle biopsy in addition to signs of rhabdomyolysis (13).

The mainstays of treatment for FAODs are provision of a low-fat, high-carbohydrate diet, as well as defect-specific fatty acid restriction/supplementation (e.g., restricting LCFAs and supplementing MCFAs in VLCADD) and carnitine and/or riboflavin supplementation. There are reports of improvement in urinary organic acid excretion, acylcarnitine levels, clinical signs, and the frequency of metabolic crises with therapy (11, 15, 20). A low-fat diet high in MCFAs was successful in treatment of the Maltese dogs with malonic aciduria (9). This therapy was not attempted in the patient described here due to the post-mortem nature of the diagnosis.

## Concluding Remarks

This paper describes the clinical, MRI, and histopathological abnormalities associated with a FAOD in a dog. Abnormalities included episodic hypoglycemia with at least a single instance of ketonuria, intermittent lactic acidosis, episodic neurological deficits progressing to persistent severe deficits, a bilaterally symmetrical multifocal necrotizing encephalopathy, multi-organ lipid accumulation (kidneys, liver, and muscle), and exacerbation by stressors including fasting. Metabolic screening, and muscle biopsy examination for lipid storage myopathy, should be performed in young dogs presenting with episodic multifocal and symmetrical encephalopathies since inborn errors of metabolism, such as FAODs and OAs, may be amenable to dietary therapeutic interventions. Enzymatic activity assays in cultured skin fibroblasts and gene sequencing are available and may be useful in identifying the specific enzymatic defect in future patients.

## Author Contributions

All listed authors (VB, JM, TD, and GS) meet all four criteria for authorship. VB and JM were responsible for all clinical aspects of the case and manuscript. TD was responsible for post-mortem examination, descriptions, interpretation, and photomicrophs/gross photographs. GS was responsible for all work and writings pertaining to muscle biopsy and metabolic screening. All authors were extensively involved in review and editing of all drafts of the manuscript.

## Conflict of Interest Statement

None of the authors have a financial or personal relationship with other persons or organizations that could inappropriately influence or bias the content of the paper.

## Footnotes

^1^Fastpanel PCR Canine Tick Bourne Panel, Antech Diagnostics, Irvine, CA, USA.

^2^Plasmalyte A, Abbott Animal Health, Abbott Park, IL, USA.

^3^Philips Achieva 1.5-T MRI, Philips Healthcare, Andover, MA, USA.

## Abbreviations

CPT, carnitine palmitoyltransferase; FAOD, fatty acid oxidation defect/disorder; LCFA, long-chain fatty acids; MADD, multiple acyl-CoA dehydrogenase deficiency; MCADD, medium-chain acyl-CoA dehydrogenase deficiency; MCD, malonyl-CoA decarboxylase; MCFA, medium-chain fatty acids; OA, organic aciduria; SCFA, short-chain fatty acids; VLCADD, very long-chain acyl-CoA dehydrogenase deficiency; VLCFA, very long-chain fatty acids.
